# GVHD prophylaxis in matched related stem cell transplantation: Why post-transplant cyclophosphamide can be recommended a study by the EBMT transplant complications working party

**DOI:** 10.1038/s41375-025-02619-1

**Published:** 2025-04-17

**Authors:** Olaf Penack, Mouad Abouqateb, Christophe Peczynski, William Boreland, Malek Benakli, Nicolaus Kröger, Igor Wolfgang Blau, Didier Blaise, Jaime Sanz, Matthias Eder, Hakan Ozdogu, Dominik Schneidawind, Annoek E. C. Broers, Gerald. G. Wulf, Alberto Mussetti, Ivan Moiseev, Charlotte Graham, Hélène Schoemans, Zinaida Peric

**Affiliations:** 1 Medical Clinic, Department for Haematology, Oncology and Tumorimmunology, Charité Universitätsmedizin Berlin, Berlin, Germany; 2 EBMT Transplant Complications Working Party, Paris, France; 3 EBMT Paris study office; Department of Haematology, Saint Antoine Hospital; INSERM UMR-S 938, Sorbonne University, Paris, France; 4 Pierre and Marie Curie Center, University of Health Sciences, Alger, Algeria; 5 University Hospital Eppendorf, Hamburg, Germany; 6 Programme de Transplantation&Therapie Cellulaire, Marseille, France; 7 Hematology Department, Hospital Universitari i Politècnic La Fe, Valencia Departament de Medicina Universitat de Valencia, CIBERONC, Instituto Carlos III, Madrid, Spain; 8 Hannover Medical School, Hannover, Germany; 9 Baskent University Hospital, Adana, Turkey; 10 University Hospital, Zürich, Switzerland; 11 Erasmus MC Cancer Institute, Rotterdam, Netherlands; 12 Universitaetsklinikum Goettingen, Goettingen, Germany; 13 Hematopoietic Cell Transplant/Cell Therapy Unit, ICO Institut Català d’Oncologia, Hospitalet de Llobregat, Barcelona, Spain; 14 RM Gorbacheva Research Institute, Pavlov University, St Petersburg, Russia; 15 Department of Hematology, Comprehensive Cancer Centre, King’s College London, London, UK; 16 University Hospitals Leuven and Department of Public Health and Primary Care, ACCENT VV, KU Leuven - University of Leuven, Leuven, Belgium; 17 Department of Haematology, University Hospital Centre Rijeka, Rijeka, Croatia

**Keywords:** Haematological diseases, Translational research

## Abstract

Rabbit anti-thymocyte globulin (rATG) reduced chronic GVHD after matched related donor (MRD) allogeneic stem cell transplantation (alloSCT) from 69% to 32% in a randomized trial and is the recommended standard in Europe. Post-transplantation Cyclophosphamide (PTCy) is an emerging alternative but lacks such solid data in MRD alloSCT. We therefore analyzed outcomes of rATG (*n* = 4140) vs. PTCy (*n* = 1069) in adult patients with hematologic malignancies undergoing MRD alloSCT between 2017 and 2021 in the EBMT database. The provided hazard ratios (HR) and P-values are adjusted for potential risk factors using multivariate analysis. Results are given at 2 years after alloSCT for all endpoints except for acute GVHD (100 days). The main difference was a lower relapse incidence after PTCy vs. rATG (26.2% vs. 32.8%; HR 0.78 [CI 95%: 0.66–0.92], *p* = 0.003). Interaction analyses confirmed the consistency of this result across different disease risk index and conditioning intensity subgroups. Other major transplant outcomes showed no significant differences: non-relapse mortality, overall survival, progression-free survival, GVHD-free relapse-free survival, incidence and severity of acute GVHD as well as chronic GVHD. In summary, PTCy results in comparable GVHD and survival outcomes but lower relapse rates compared to rATG. We conclude that PTCy can be recommended in MRD alloSCT.

## Introduction

A major complication of allogeneic stem cell transplantation (alloSCT) is the occurrence of chronic graft-versus-host disease (cGVHD) leading to often massively reduced quality of life as well as to increased mortality. The prevention strategies of GVHD are currently changing. In transplantations from matched related stem cell donors (MRD) rabbit anti-thymocyte globulin (rATG, also termed anti-T-cell globulin or anti-T-lymphocyte globulin; products: Grafalon® or Thymoglobulin®) is the standard of care in Europe to decrease the cGVHD risk [1]. Cyclophosphamide given after alloSCT (post-transplant Cyclophosphamide, PTCy) is increasingly used in the MRD setting but published data has not been solid enough to support standard use in this setting [1]. Comparative data from larger datasets of PTCy vs. rATG in the MRD setting are missing, preventing evidence based clinical decision making.

The recommendation of the European Society for Blood and Marrow Transplantation (EBMT) to primarily use rATG in the MRD setting is mainly based on strong evidence from a Phase III randomized trial [2, 3]. The inclusion of rATG resulted in a significantly lower rate of cGVHD (32%) as compared to standard of care without rATG (69%). In addition, the rate of a composite end point of chronic GVHD–free survival and relapse free survival was higher in the rATG arm (37% vs. 17%). At five years follow up, fewer patients remained on immunosuppressive medications in the rATG arm (10% vs. 29%).

Data for PTCy use in MRD is limited as no randomized trial specifically addressed this question. Four randomized studies comparing PTCy prophylaxis vs. no PTCy (without ATG) in alloSCT also enrolled recipients of MRD alloSCT [4–7]. The incidence of both aGVHD and cGVHD was significantly reduced with PTCy in the patients enrolled by the CTN 1703 trial [4] as well as in non-randomized studies [8–10] but the relatively low percentage of MRD alloSCTs in these studies limit their significance for MRD alloSCTs. There is to date no specific direct evidence comparing PTCy vs. rATG prophylaxis in MRD alloSCT, since the reported populations always included both MRD and MUD (a meta-analysis [11], a randomized trial [12], and retrospective studies [13, 14]).

To address this knowledge gap, we analyzed outcomes of PTCy vs. rATG prophylaxis in adult patients with hematologic malignancies undergoing a first alloSCT from MRD between Jan 2017 and December 2021 in the database of the EBMT.

## Patients and methods

### Study design and data collection

This is a retrospective multicenter analysis using the data set of the EBMT registry. The EBMT is a voluntary working group of more than 600 transplant centers which are required to report regular follow up on all consecutive stem cell transplantations. Audits are routinely performed to determine the accuracy of the data. The study was planned and approved by the Transplant Complications Working Party of the EBMT. All patients gave their written informed consent to use their personal information for research purposes. The study was conducted in accordance with the Declaration of Helsinki and Good Clinical Practice guidelines.

Eligibility criteria for this analysis included patients older than 18 years of age at alloSCT with hematologic malignancies (acute lymphoblastic leukemia, acute myeloid leukemia, lymphoma, chronic lymphocytic leukemia, myelodysplastic syndrome or myeloproliferative neoplasms), who underwent a first alloSCT from a matched related donor (MRD, matched sibling donor), from a peripheral blood or bone marrow stem cells source, between Jan 2017 and December 2021 in the database of the EBMT. Only patients receiving either rATG or PTCy based GVHD prophylaxis were included. Additionally, patients with more than one previous autologous transplantation, ex-vivo T-cell depletion, a combination of rATG and PTCy or use of Alemtuzumab (Campath) were excluded from the study. Data collected included recipient and donor characteristics (age, sex, cytomegalovirus serostatus and Karnofsky performance status score), diagnosis and status at transplant and transplant-related factors, including conditioning regimen, stem cell source and GVHD prophylaxis. GVHD grading was performed according to published criteria for acute GVHD [15] and chronic GVHD [16]. For the purpose of this study, all necessary data were collected according to the EBMT guidelines, using the EBMT Minimum Essential Data forms.

### Statistical analysis

Median values and interquartile ranges (IQR), and minimum and maximum values were used to describe quantitative variables; frequency and percentage were used for categorical variables. Main patient-, disease-, and transplant-related characteristics were compared using Pearson’s Chi-squared test for categorical variables, and the Wilcoxon rank sum test for quantitative variables between the two groups.

Study endpoints were non-relapse mortality (NRM), overall survival (OS), progression-free survival (PFS), relapse incidence (RI), GVHD-free/relapse-free survival (GRFS), and incidence and severity of acute and chronic GVHD. The initial time was the date of transplant for all endpoints. NRM was defined as death without relapse/progression, PFS was defined as survival without relapse or progression, RI was defined as disease recurrence, GRFS was defined as survival without incidence of relapse, or grade III–IV acute GVHD, or extensive chronic GVHD. Probabilities of OS, PFS and GRFS were calculated using the Kaplan-Meier method. Cumulative incidence was used to estimate NRM, RI, as well as acute and chronic GVHD in a competing risk setting, where death and relapse were considered as competing risk as appropriate [17]. Multivariate analyses were performed using the Cox cause-specific proportional-hazards model for all endpoints. All known potential risk factors, and variables differing significantly across the groups were included in the multivariate models: patient age at transplant, year of transplant, patient and donor sex at birth, donor to patient CMV combination, Disease Risk Index (DRI), Karnofsky Performance Status (KPS), any level of total body irradiation (TBI), conditioning intensity (RIC vs. MAC). Center effect was considered by introducing a random effect or ‘frailty’ into all models. Results were expressed as the hazard ratio (HR) with the 95% confidence interval (95% CI). We conducted interaction analyses to determine whether the effect of rATG vs. PTCy on relapse incidence differed across clinically relevant subgroups, we added interaction terms to the main Cox models separately for PTCy vs. ATG with disease risk index disease risk index (DRI: high vs. low and intermediate vs. low, considering disease entities and remission status prior to transplant) and with conditioning intensity (myeloablative conditioning [MAC] vs. reduced intensity conditioning [RIC]), subgroup-specific estimates were derived from the analysis for illustrative purposes only. All tests were 2-sided with a type 1 error rate fixed at 0.05. Statistical analyses were performed with R 4.3.0 software (R Development Core Team, Vienna, Austria) packages.

## Results

### Patient characteristics

The baseline characteristics of the study population are presented in Table 1. A total of 5209 patients were included, from which 4140 (79.5%) received rATG, and 1069 (21.5%) received PTCy as GVHD prophylaxis.

**Table 1:** Baseline patient-, donor- and transplant-related characteristics by graft-versus-host disease prevention strategy.

	ATG based (*N* = 4140)	PTCy based (*N* = 1069)	*p* value
**Medium follow up time years (CI 95%)**	2.2 (2.1–2.3)	2 (1.9–2.1)	
**Patient Sex at Birth**			
Male	2426 (58.6%)	628 (58.7%)	0.93
Female	1714 (41.4%)	441 (41.3%)	
**Age at Transplant, yrs**			
median [Q1, Q3]	54.3 (43.7, 61.1)	51.3 (39.0, 59.9)	<0.01
[Min, Max]	18.0–77.7	18.0–75.6	
**Donor Age**			
median [Q1, Q3]	52.3 (42.9, 59.6)	49.1 (38.2, 57.6)	<0.01
[Min, Max]	18.0–77.0	18.0–75.5	
**Karnofsky**			<0.01
<90	1171 (29.7%)	249 (24.2%)	
>= 90	2775 (70.3%)	778 (75.8%)	
Missing count	194	42	
**HCT Comorbidity Index**			<0.01
0	2151 (59.8%)	528 (52.5%)	
1–2	720 (20.0%)	228 (22.7%)	
>=3	726 (20.2%)	250 (24.9%)	
Missing count	543	63	
**DRI**			<0.01
Low	216 (5.2%)	104 (9.7%)	
Int	2829 (68.3%)	687 (64.3%)	
High	938 (22.7%)	242 (22.6%)	
Very high	157 (3.8%)	36 (3.4%)	
**Hematological Malignancies**			Not done
AML	2255 (54.5%)	458 (42.8%)	
MDS	570 (13.8%)	120 (11.2%)	
ALL	413 (10.0%)	185 (17.3%)	
NHL	308 (7.4%)	148 (13.8%)	
MPN	392 (9.5%)	51 (4.8%)	
MDS & MPN	142 (3.4%)	35 (3.3%)	
Hodgkin’s Lymphoma	60 (1.4%)	72 (6.7%)	
**Transplant Year**			<0.01
2017	903 (21.8%)	147 (13.8%)	
2018	890 (21.5%)	194 (18.1%)	
2019	848 (20.5%)	233 (21.8%)	
2020	719 (17.4%)	236 (22.1%)	
2021	780 (18.8%)	259 (24.2%)	
**Myeloablative Conditioning**			
No	1865 (45.5%)	472 (44.6%)	=0.61
Yes	2234 (54.5%)	586 (55.4%)	
Missing count	41	11	
**TBI**			< 0.011
No	3629 (87.7%)	800 (74.8%)	
Yes	511 (12.3%)	269 (25.2%)	
Cell Source			Not done
BM	159 (3.8%)	131 (12.3%)	
Cell SourcePB	3981 (96.2%)	938 (87.7%)	
**GVHD Prevention Regimen**			
CSA + MTX	1693 (40.9%)	24 (2.2%)	
CSA + MMF	1335 (32.2%)	209 (19.6%)	
CSA only	755 (18.2%)	200 (18.7%)	
TACRO + SIRO + MMF	235 (5.7%)	285 (26.7%)	
TACRO + SIRO	33 (0.8%)	199 (18.6%)	
TACRO + MTX	34 (0.8%)	2 (0.2%)	
No additional drugs or other regimen	55 (1.4%)	150 (14.0%)	

Overall, the majority of patients were transplanted for acute leukemia (63.6%), myelodysplastic syndrome (MDS) (13.2%), lymphoma (11.3%) or myeloproliferative neoplasm (MPN) (8.5%). A high proportion of patients had an intermediate Disease Risk Index (DRI, 67.5%), and myeloablative conditioning (MAC) was more frequently performed (54.7%) than reduced intensity conditioning (RIC). Detailed data on conditioning regimens is given in Supplementary Table 1.

We were interested in the type and dosage of rATG used. Supplementary Table 2 shows the dosage information (25.4% missing data) as well as the product type. Supplementary Fig. 1 shows the distribution of cumulative ATG Dosages. Patients in the rATG group were older, with a median age of 54.3 years (IQR (43.7, 61.1)) vs. 51.3 years in the PTCy group (IQR 39.0, 59.9; *p* < 0.01), with a similar proportion of males (58.6% in rATG vs. 58.7% in PTCy, *p* = 0.93), along with a significantly lower use of TBI (12.3% vs. 25.2%, *p* < 0.01). Also, the disease risk index and the Karnofsky performance score were lower and the year of transplant was more recent in the PTCy group (Table 1). The donor age was also lower in the PTCy group (Table 1 and Supplementary Fig. 2, *p* < 0.01). The remaining parameters were balanced between the two groups. Median follow up was 2.2 years (CI95%: 2.1–2.3) in the rATG vs. 2 years (CI95%: 1.9–2.1) in the PTCy arm. More detailed information is given in Table 1.

### Survival, NRM and RI

**Fig. 1:**
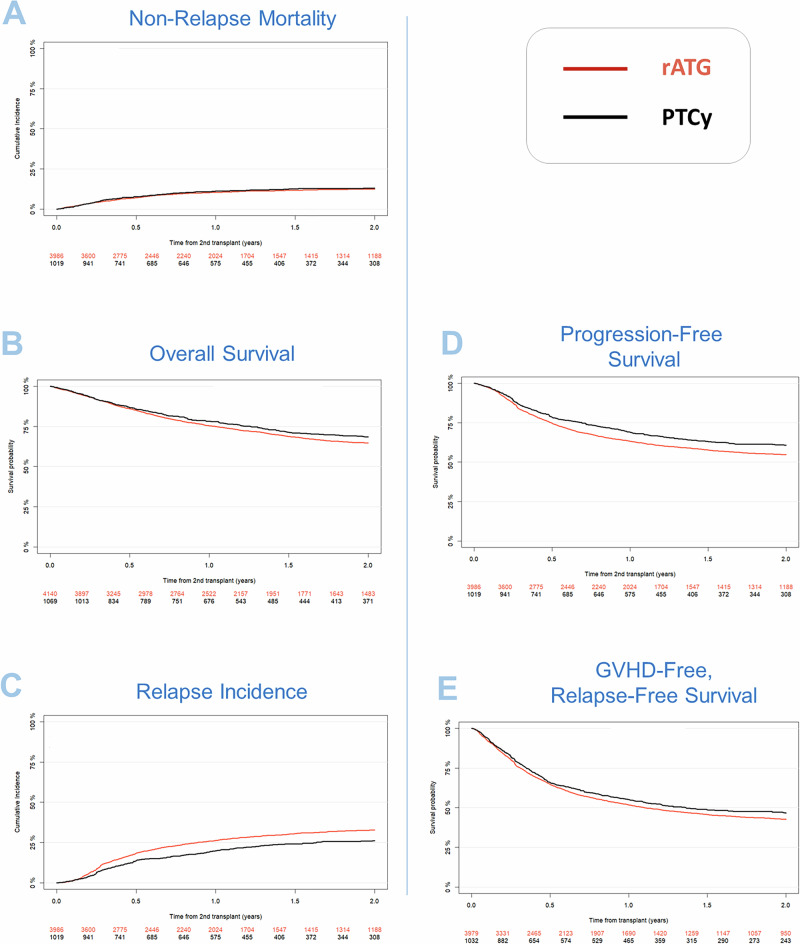
Survival and relapse outcome parameters. **A** NRM, **B** Overall survival, **C** Relapse incidence, **D** Progression-free survival and **E** GVHD-free relapse-free survival. Presented as Kaplan-Meier survival curves and cumulative incidences curves.

Univariate outcomes are shown in Fig. 1 and Table 2. The results of the multivariate analyses are summarized in Table 3 as well as the Supplementary Tables 3 and 4. The *P*-values and hazard ratios (HR) presented in the following results section are derived from the multivariate analysis.

**Table 2:** Incidence of major transplant outcome variables.

	rATG (CI 95%)	PTCy (CI 95%)
**Non-relapse mortality**	12.5% (11.4–13.6)	13.1% (10.9–15.4)
**Relapse incidence**	32.8% (31.2–34.4)	26.2% (23.2–29.2)
**Overall survival**	64.8% (63.1–66.4)	68.5% (65.2–71.6)
**Progression-free survival**	54.7% (53–56.4)	60.7% (57.3–64)
**GVHD-free and Relapse-free survival**	42.7% (41–44.4)	46.5% (43.1–49.8)
**Acute GVHD-II/IV**	19.5% (18.2–20.7)	21.4% (18.9–24)
**Acute GVHD-III/IV**	7.7% (6.9–8.6)	7.6% (6–9.3)
**Chronic GVHD**	28.9% (27.4–30.5)	34.4% (31.2–37.6)
**Extensive chronic GVHD**	12.6% (11.5–13.8)	16.1% (13.7–18.7)

All outcomes except acute GVHD are given at two years. Acute GVHD is given at day +100 after alloSCT.

**Table 3:** Multivariate analysis. Hazard ratios (HR) are given for PTCy with rATG being the comparator.

	HR (95% CI)	*p*-value
**Relapse incidence**	0.78 (0.66 to 0.92)	**0.003**
**Non-relapse mortality**	1.22 (0.95 to 1.57)	0.12
**Overall survival**	0.99 (0.85 to 1.16)	0.92
**Progression-free survival**	0.89 (0.78 to 1.03)	0.11
**GVHD-free and Relapse-free survival**	0.94 (0.82 to 1.07)	0.32
**Acute GVHD-II/IV**	1.10 (0.88 to 1.37)	0.39
**Acute GVHD-III/IV**	1.02 (0.74 to 1.39)	0.93
**Chronic GVHD**	1.01 (0.83 to 1.23)	0.90
**Extensive chronic GVHD**	0.97 (0.72 to 1.31)	0.85

All known potential risk factors, and variables differing significantly across the groups were included in the multivariate models: patient age at transplant, year of transplant, patient and donor gender, donor to patient CMV combination, Disease Risk Index (DRI), Karnofsky Performance Status (KPS), any level of total body irradiation (TBI), conditioning intensity (RIC vs. MAC). Center effect was considered by introducing a random effect or ‘frailty’ into all models.

**Fig. 2:**
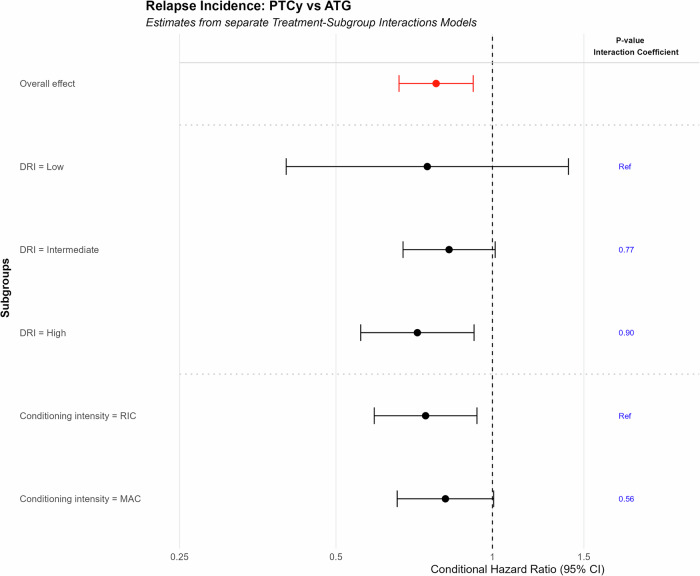
Interaction analyses of PTCy vs. rATG on relapse incidence: Hazard Ratios and Interaction tests for disease risk index (DRI) and conditioning intensity subgroups. The effect PTCy vs. rATG on relapse rates is consistent across the subgroups (*p* > 0.05).

The main difference we found in this study was in relapse incidence (RI). RI was lower in the PTCy arm compared to rATG (2-year incidence: 26.2% vs. 32.8%; HR: 0.78 [95% CI 0.66–0.92], *p* = 0.003) compared to rATG. To assess whether the effect of PTCy vs. rATG on relapse incidence differed across DRI and conditioning intensity risk subgroups, we conducted interaction analyses (Fig. 2). No interaction term was found statistically significant (*p*-values >> 0.05), supporting our primary finding of a lower relapse rate with PTCy compared to rATG and its consistency across the examined subgroups (overall HR: 0.78 [95% CI 0.66–0.92], *p* = 0.003). Figure 2 displays the subgroups estimates for illustrative purposes, along with *p*-values for the corresponding interactions. Since over 45% of patients in the PTCy group received Tacrolimus/Sirolimus ± MMF we explored additional adjustments on additional use of Tacrolimus/Sirolimus ± MMF and results remain consistent (HR PTCy vs. rATG: 0.73 [95%CI: 0.61–0.88]; *p* < 0.01). Sensitivity analysis excluding patients without additional use of Tacrolimus/Sirolimus ± MMF (from both groups) also showed significant results in favor of PTCy (HR PTCy vs. rATG 0.71 (95% CI: 0.58–0.88); *p* = 0.002). Testing for an interaction between years of transplantation and relapse, and between total body irradiation (TBI) and relapse, indicated consistent results in favor of PTCy for relapse incidence across different years of alloSCT and TBI groups.

We found no statistically significant differences in survival related outcomes. Patients receiving PTCy showed a non-significant trend toward higher NRM compared to rATG (2-year incidence: 13.1% vs. 12.5%; HR: 1.22 [95% CI 0.95–1.57], *p* = 0.12). The 2-year incidence of overall survival was 68.5% for PTCy vs. 64.8% for rATG; HR: 0.99 [95% CI 0.85–1.16], *p* = 0.92. PFS showed a non-significant positive tendency in the PTCy arm vs. the rATG arm (2-year incidence: 60.7% vs. 54.7%; HR: 0.89 [95% CI 0.78–1.03], *p* = 0.11).

The causes of death are given in Table 4. Relapse of the underlying malignancy was the most frequent cause of death, accounting for 61.6% of total deaths in both arms, followed by NRM causes: infections 14.2%, GVHD 11.6% and other alloSCT-related causes 6.2% of total deaths. Secondary malignancies contributed to 1% of total deaths.

**Table 4:** Causes of death in both cohorts. Absolute numbers and percentages are given.

Causes	ATG, *N* = 1377	PTCY, *N* = 309
Categories, *n*(%)		
Original Disease	846 (62.9%)	171 (56.1%)
Infection	184 (13.7%)	51 (16.7%)
GVHD	150 (11.1%)	41 (13.4%)
Other HSCT related	82 (6.1%)	23 (7.5%)
Other	72 (5.4%)	13 (4.3%)
Secondary malignancy	12 (0.9%)	6 (2%)
Unknown	31	4

### Incidence of acute and chronic GVHD, and GVHD-free relapse free survival (GRFS)

**Fig. 3:**
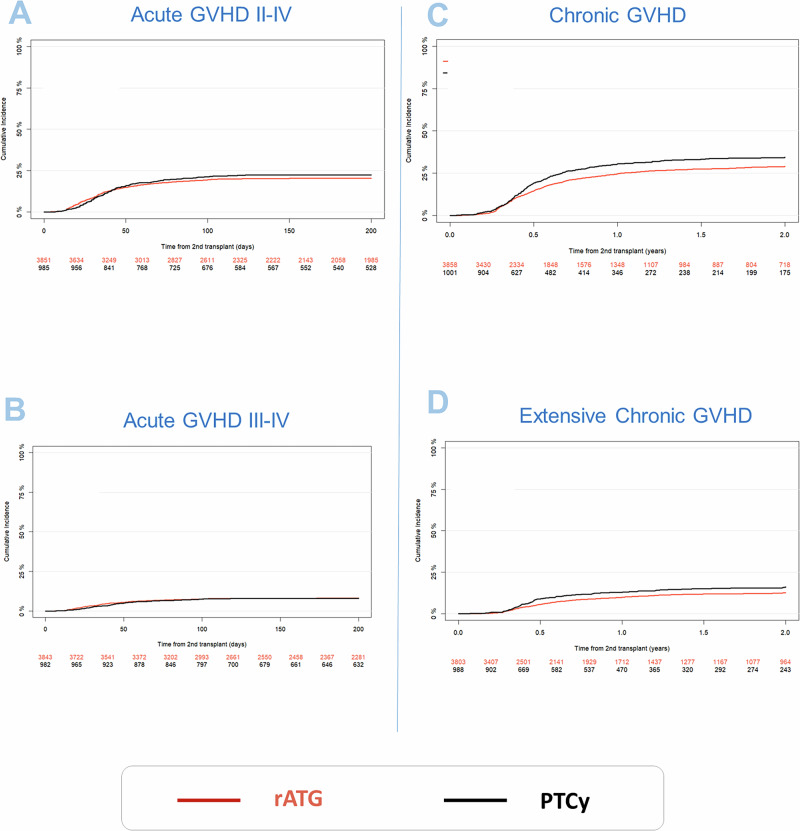
GVHD outcome parameters. **A** Acute GVHD grades II–IV; **B** Acute GVHD grades III-IV, **C** Chronic GVHD all grades and **D** Extensive chronic GVHD - Cumulative incidence curves are shown.

Univariate outcomes are shown in Fig. 3 and Table 2. The results of the multivariate analyses are summarized in Table 3. We detected no major differences regarding the incidence or severity of acute and chronic GVHD. The incidence of acute GVHD grades II-IV in patients receiving PTCy, compared to those receiving ATG did not differ significantly: (100-day incidence: 21.4% vs. 19.5%; HR: 1.1 [95% CI 0.88 to 1.37], *p* = 0.39). Similarly, for severe acute GVHD grades III-IV (100-day incidence: 7.6% for PTCy vs. 7.7% for rATG; HR: 1.02 [95% CI 0.74–1.39], *p* = 0.93). The 2-year incidence of overall chronic GVHD was 34.4% in the PTCy arm vs. 28.9% in rATG; HR: 1.01 [95% CI 0.83–1.23], (*p* = 0.9). Extensive chronic GVHD at 2-year was at 16.1.9% for PTCy vs. 12.6% for rATG (HR: 0.97 [95% CI 0.72–1.31], *p* = 0.85).

GRFS at 2 y was 46.5% in the PTCy arm compared to 42.7% in the rATG arm; HR: 0.94 [95% CI 0.82 to 1.07], (*p* = 0.32).

## Discussion

In MRD alloSCT, rATG or PTCy are often used as part of the GVHD prophylaxis strategy. In Europe, it has been standard of care to use rATG in MRD alloSCTs with a high GVHD risk [1]. In the USA, the results of the CTN 1703 and CTN 1203 randomized trials [4, 5], demonstrating a benefit of PTCy vs. no T-cell depletion mainly in matched unrelated donor (MUD) alloSCT, led to a widespread use of PTCy also in the MRD setting. The present study was designed to help answering the question if PTCy or rATG should be the preferred option in this setting. In recipients of MRD alloSCT, we found that PTCy prophylaxis vs. rATG prophylaxis was associated with similar NRM, overall survival and GVHD incidences. The main difference we detected was a lower incidence of relapse in alloSCT recipients receiving PTCy that was independent from the relapse risk index and from conditioning intensity. This finding is in line with the results of our recent analysis in the MUD setting, where PTCy also resulted in a lower relapse incidence as compared to rATG [18]. The most likely explanation is the stronger anti-tumor activity of Cyclophosphamide as compared to rATG, which also has some anti-tumor efficacy in hematologic malignancies [19]. Because of the lower relapse rate after PTCy use it will be important to find out if patients with specific tumor entities experience greater benefit from PTCy use. Future studies will need to focus on the differential impact of PTCy vs. rATG on relapse rates in different tumor entities (e.g. lymphoid malignancies vs. myeloid neoplasms) led by disease specific working parties with access to large sets of patient data (e.g. EBMT or Center for International Blood & Marrow Transplant Research [CIBMTR]).

One important finding from this study is that most major transplant outcomes parameters, such as NRM, overall survival and GVHD incidences, were not significantly different in the PTCy vs. the rATG group. This was somehow surprising since our recent analyses in both matched unrelated donors (MUD) and mismatched unrelated donors (MMUD) found a significantly lower NRM, lower GVHD incidence as well as better overall survival after PTCy use in alloSCT [18, 20]. We can only speculate why the results in MRD differ so strikingly from the MUD and MMUD settings. However, we believe that the more extensive allo-activation of T cells early after MUD/MMUD and consequently more efficacious depletion of allo-activated T cells by PTCy may play a critical role [21].

The limitations of the current study are typical to retrospective real-world analyses: there is a low granularity, a risk of underreporting and there are potential confounding factors. We observed significant differences in baseline characteristics, with patients in the rATG group being older, having a higher disease risk index, a lower performance index and having received LESS total body irradiation. Another confounding factor of the real-world data is that there was a wide variety of immunosuppressive regimens given in addition to rATG or PTCY. Also, different formulations and dosing of rATG were used. Of note, our observation period is relatively short because PTCy prophylaxis is a relatively recent practice, which could also play in favor of the better outcome of these patients. Therefore, we are unable to draw final conclusions regarding long term outcome and the occurrence of late effects. One important issue is the occurrence of secondary malignancies: more long-term follow up will be needed to answer the question if long-term effects differ between PTCy and rATG. Another aspect is the measurement of minimal residual disease (MRD), which is clinically relevant. Due to missing values for MRD (in 73% of the observations) in the EBMT database and the heterogeneity of methods used to measure MRD, we were unable to include data. Finally, a limitation is that we included the Disease Risk Index (DRI) into the multivariate analysis but did not sub-analyze the differential effects of PTCy and rATG on specific hematologic malignancies, such as acute myeloid leukemia versus lymphoid neoplasms. This did not fall into the scope of this analysis and may be attempted by the disease-specific EBMT working parties.

To further improve outcomes in the future, PTCy regimens could be optimized and/or could be administered in combination with rATG. Optimization of the PTCy dosing schedule and combination with immunosuppressive agents may improve efficacy [22]. In murine models lower PTCy doses prevented fatal GVHD [23] and a clinical study found faster engraftment, lower mucositis incidence and a lower infection rate when 25 mg/kg/d Cyclophosphamide was used compared to the standard dose of 50 mg/kg/d [24]. A combination of rATG and PTCy has been first tested in haploidentical SCT (haploSCT) [25–27]. In a recent publication the EBMT reported patients after haploSCT receiving either PTCy (*n* = 2999), rATG (*n* = 358), or a combination of rATG and PTCy (*n* = 292) as GVHD prophylaxis [28]. The combination of PTCy and rATG was associated with reduced rates of acute GVHD and did not affect other major transplant outcome parameters. Since there is no solid evidence published in MRD on a combination of rATG with PTCy this could be a focus area for future clinical research.

In summary, PTCy resulted in comparable GVHD and survival outcomes but lower relapse rates as compared to rATG in our study. We conclude that PTCy can be recommended in MRD alloSCT. However, certain patient populations, for instance those with high cardiovascular risks, might not be ideal candidates for PTCy because of its cardiac toxicity [29]. There is the need to collect more data on long term outcome in this setting [30].

## Supplementary information

Supplementary material

## Data Availability

Individual participant data will not be shared because patients agreed to data sharing with EBMT as well as with publication of results, but not to share data with third parties.
