# Supplementary material for: GVHD prophylaxis in matched related stem cell transplantation: Why post-transplant cyclophosphamide can be recommended a study by the EBMT transplant complications working party
Source: Leukemia. 2025 Apr 17;39(6):1503–11. doi: 10.1038/s41375-025-02619-1 (PMC12133576; doi:10.1038/s41375-025-02619-1)
Supplement: Supplementary file 1 — Supplementary material [file 41375_2025_2619_MOESM1_ESM.docx]

**Supplemenatary Figure 1**. Distribution of cumulative ATG Dosages.


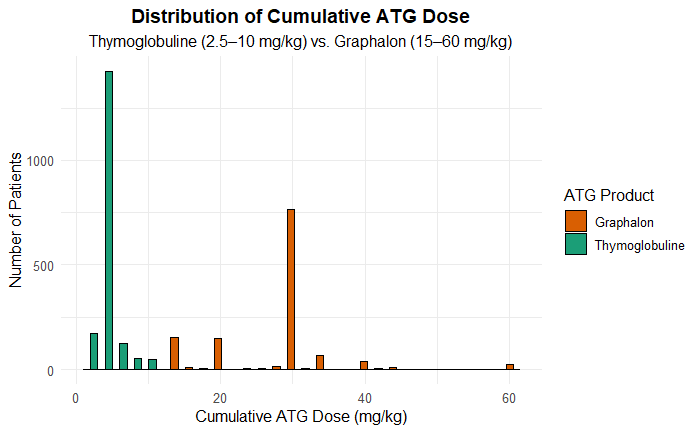


**Supplementary Figure 2.** Distribution of donor age.

**
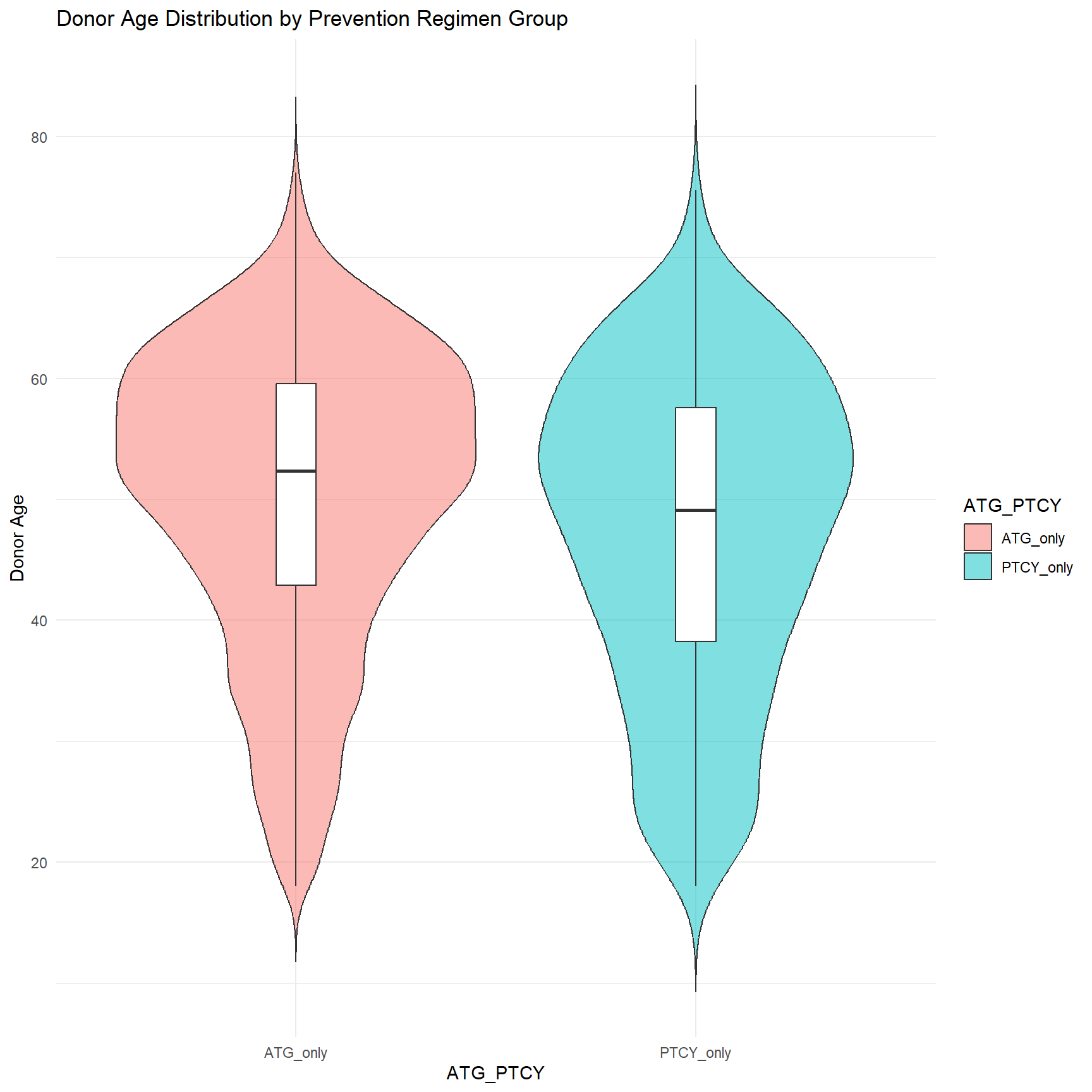
**

**Supplementary Table 1.** Conditioning regimens.

| **Conditioning Regimen** | **Overall Count** | **Conditioning Drugs** | **ATG_only** | **PTCY_only** |
| --- | --- | --- | --- | --- |
| BuFlu based | 2364 | BuFlu | 1912 (95.0%) | 316 (90.0%) |
|  |  | BuCy+Flu | 26 (1.3%) | 12 (3.4%) |
|  |  | BuFlu+Other | 75 (3.7%) | 23 (6.6%) |
| BuFluThio based | 652 | BuFlu+Thio | 339 (88.7%) | 254 (94.1%) |
|  |  | BuCy+Flu+Eto+Thio | 36 (9.4%) | 5 (1.9%) |
|  |  | BuFlu+Thio+Ritux | 5 (1.3%) | 1 (0.4%) |
|  |  | BuCy+Flu+Thio | _ | 4 (1.5%) |
|  |  | BuFlu+Thio+Other | 2 (XX%) | 6 (XX%) |
| FluMel based | 348 | FluMel | 164 (57.5%) | 38 (60.3%) |
|  |  | FluMel+BCNU | 83 (29.1%) | 5 (7.9%) |
|  |  | FluMel+Thio | 27 (9.5%) | 14 (22.2%) |
|  |  | FluMel+Other | 11 (3.9%) | 6(9.52%) |
| TreoFlu based | 332 | Treo+Flu | 196 (73.7%) | 29 (43.9%) |
|  |  | Treo+FluMel | 27 (10.2%) | 31 (47.0%) |
|  |  | Treo+Flu+Thio | 33 (12.4%) | 2 (3.0%) |
|  |  | Treo+Flu+Other | 12 (3.7%) | 3 (4.5%) |
| Flamsa chemo | 301 | BuCy+Flamsa | 151 (50.8%) | _ |
|  |  | Bu+Flamsa | 99 (33.3%) | 2 (50.0%) |
|  |  | Treo+Cy+Flamsa | 21 (7.1%) | 1 (25.0%) |
|  |  | Flamsa Only | 2 (0.7%) | 1 (25.0%) |
|  |  | Flamsa+Other | 23 (7.7%) | _ |
| BuCy based | 234 | BuCy | 190 (85.2%) | 11 (100.0%) |
|  |  | BuCy+Arac | 28 (12.6%) | _ |
|  |  | BuCy+Other | 5 (2.2%) | _ |
| Bu+Other | 53 | Bu+Thio | 19 (42.2%) | 3 (37.5%) |
|  |  | Bu+Clofa | 14 (31.1%) | 2 (25.0%) |
|  |  | Bu Only | 4 (8.9%) | 2 (25.0%) |
|  |  | Bu+Arac+Clofa | 3 (6.6%) | _ |
|  |  | Bu+Other | 5 (11%) | 1 (12.5%) |
| FluCyThio based | 39 | Cy+Flu+Thio | 26 (78.8%) | 6 (100.0%) |
|  |  | Cy+Flu+Thio+Ritux | 5 (15.2%) | _ |
|  |  | Cy+Flu+Eto+Thio | 2 (6.0%) | _ |
| FluCy based | 19 | Cy+Flu | 14 (93.3%) | 4 (100.0%) |
|  |  | Cy+Flu+Arac | 1 (6.7%) | _ |
| Other | 60 | Other | 46 | 14 |

**Supplemenatry Table 2**. rATG dosage by product types in mg/KG body weight.

| **ATG dose, mg/Kg** | **Graphalon (N=1268)** | **Thymoglobuline (N=1820)** | **Total (N=3088)** |
| --- | --- | --- | --- |
| median [Q1, Q3] | 30.0 (20.0, 30.0) | 5.0 (5.0, 5.0) | 5.0 (5.0, 30.0) |
| [Min, Max] | 15.0 - 60.0 | 1.5 - 10.0 | 1.5 - 60.0 |

#### **Supplementary table 3**. Multivariate analysis of survival and relapse related outcomes

|  | **Non Relapse Mortality NRM** | | **Overall Survival OS** | | **Relapse Incidence RI** | | **Progression Free Survival PFS** | | **GRFS** | |
| --- | --- | --- | --- | --- | --- | --- | --- | --- | --- | --- |
|  | HR (95% CI)*1* | p-val*2* | HR (95% CI)*1* | p-val*2* | HR (95% CI)*1* | p-val*2* | HR (95% CI)*1* | p-val*2* | HR (95% CI)*1* | p-val*2* |
| **GVHD Prophylaxis Regimen** |  |  |  |  |  |  |  |  |  |  |
| PTCy vs. ATG | 1.22 (0.95-1.57) | 0.12 | 0.99 (0.85-1.16) | 0.92 | 0.78 (0.66-0.92) | **0.003**** | 0.89 (0.78-1.03) | 0.11 | 0.94 (0.82-1.07) | 0.32 |
| **Patient Donor Sex:** Female_to_Male vs Ref | 1.34 (1.11-1.60) | **0.002**** | 1.10 (0.98-1.23) | 0.10 | 0.98 (0.87-1.11) | 0.79 | 1.08 (0.97-1.19) | 0.15 | 1.07 (0.98-1.17) | 0.12 |
| **Age at Transplant,** 5yrs inc | 1.17 (1.13-1.23) | **<0.001***** | 1.08 (1.05-1.10) | **<0.001***** | 0.98 (0.96-1.00) | 0.074 | 1.03 (1.00-1.05) | **0.015*** | 1.03 (1.02-1.05) | **<0.001***** |
| **Karnofsky** ( >= 90 vs. <90) | 0.73 (0.60-0.89) | **0.002**** | 0.73 (0.65-0.81) | **<0.001***** | 0.90 (0.79-1.02) | 0.088 | 0.85 (0.76-0.94) | **0.002**** | 0.84 (0.76-0.92) | **<0.001***** |
| **Disease Risk Index (DRI)** [Ref: Low] |  |  |  |  |  |  |  |  |  |  |
| Int | 0.84 (0.58-1.22) | 0.36 | 1.18 (0.92-1.53) | 0.20 | 1.51 (1.13-2.01) | **0.005**** | 1.26 (1.00-1.58) | **0.048*** | 1.19 (0.98-1.44) | 0.079 |
| High_VeryHigh | 0.96 (0.65-1.42) | 0.84 | 2.20 (1.70-2.86) | **<0.001***** | 2.98 (2.22-4.00) | **<0.001***** | 2.16 (1.71-2.73) | **<0.001***** | 1.82 (1.49-2.22) | **<0.001***** |
| **Transplant Year, yrs** | 1.00 (0.94-1.07) | 0.88 | 0.99 (0.95-1.03) | 0.73 | 0.98 (0.94-1.02) | 0.26 | 0.99 (0.95-1.02) | 0.39 | 0.98 (0.95-1.01) | 0.17 |
| **Cell Source** PB vs. BM | 1.43 (0.88-2.34) | 0.15 | 1.05 (0.81-1.35) | 0.73 | 1.02 (0.79-1.31) | 0.89 | 1.10 (0.87-1.37) | 0.43 | 1.02 (0.84-1.25) | 0.81 |
| **TBI use** | 0.99 (0.75-1.30) | 0.92 | 1.01 (0.86-1.18) | 0.94 | 1.01 (0.86-1.19) | 0.86 | 1.01 (0.88-1.16) | 0.89 | 1.05 (0.92-1.18) | 0.48 |
| **Myeloablative Conditioning** | 0.97 (0.80-1.18) | 0.77 | 0.95 (0.84-1.06) | 0.34 | 0.81 (0.72-0.91) | **<0.001***** | 0.85 (0.77-0.94) | **0.002**** | 0.86 (0.79-0.95) | **0.002**** |
| **Donor-Patient CMV positivity (Ref:** Neg-Neg**)** |  |  |  |  |  |  |  |  |  |  |
| Neg-Pos | 0.95 (0.70-1.30) | 0.76 | 0.91 (0.76-1.08) | 0.28 | 0.87 (0.72-1.05) | 0.14 | 0.89 (0.76-1.05) | 0.16 | 0.89 (0.77-1.02) | 0.090 |
| Pos-Neg | 1.14 (0.81-1.60) | 0.45 | 1.01 (0.83-1.23) | 0.91 | 1.02 (0.83-1.26) | 0.82 | 1.05 (0.88-1.25) | 0.57 | 0.93 (0.80-1.09) | 0.38 |
| Pos-Pos | 1.21 (0.95-1.55) | 0.12 | 1.10 (0.95-1.26) | 0.20 | 1.00 (0.87-1.16) | 0.96 | 1.06 (0.94-1.20) | 0.37 | 1.00 (0.90-1.11) | >0.99 |

| *1* HR = Hazard Ratio, CI = Confidence Interval |
| --- |
| *2* *p<0.05; **p<0.01; ***p<0.001 |

#### **Supplemenatry Table 4**. Multivariate analysis of GVHD related outcomes

|  | **CGVH** | | **CGVH EXT** | | **aGVH-II/IV** | | **aGVH-III/IV** | |
| --- | --- | --- | --- | --- | --- | --- | --- | --- |
|  | HR (95% CI)*1* | p-val*2* | HR (95% CI)*1* | p-val*2* | HR (95% CI)*1* | p-val*2* | HR (95% CI)*1* | p-val*2* |
| **GVHD Prophylaxis Regimen** |  |  |  |  |  |  |  |  |
| PTCy vs. ATG | 1.01 (0.85-1.20) | 0.90 | 0.97 (0.75-1.26) | 0.85 | 1.10 (0.91-1.33) | 0.39 | 1.02 (0.74-1.39) | 0.93 |
| **Patient Donor Sex:** Female_to_Male vs Ref | 1.11 (0.98-1.27) | 0.10 | 1.13 (0.94-1.37) | 0.20 | 1.20 (1.04-1.38) | **0.012*** | 1.04 (0.82-1.30) | 0.77 |
| **Age at Transplant,** 5yrs inc | 1.05 (1.03-1.08) | **<0.001***** | 1.08 (1.04-1.13) | **<0.001***** | 1.00 (0.97-1.03) | 0.96 | 1.04 (0.99-1.08) | 0.15 |
| **Karnofsky** ( >= 90 vs. <90) | 0.92 (0.80-1.05) | 0.23 | 0.82 (0.67-1.00) | 0.060 | 1.00 (0.86-1.16) | 0.95 | 0.76 (0.60-0.96) | **0.022*** |
| **Disease Risk Index (DRI)** [Ref: Low] |  |  |  |  |  |  |  |  |
| Int | 1.11 (0.86-1.43) | 0.45 | 1.13 (0.76-1.68) | 0.56 | 1.07 (0.80-1.43) | 0.67 | 1.16 (0.70-1.91) | 0.58 |
| High_VeryHigh | 1.06 (0.81-1.40) | 0.66 | 0.99 (0.64-1.52) | 0.96 | 1.22 (0.90-1.66) | 0.21 | 1.46 (0.87-2.47) | 0.15 |
| **Transplant Year, yrs** | 0.96 (0.92-1.00) | 0.065 | 0.98 (0.92-1.05) | 0.58 | 1.00 (0.95-1.04) | 0.85 | 0.96 (0.89-1.03) | 0.25 |
| **Cell Source** PB vs. BM | 1.04 (0.79-1.35) | 0.81 | 1.16 (0.76-1.77) | 0.51 | 1.02 (0.76-1.37) | 0.89 | 0.72 (0.47-1.12) | 0.15 |
| **TBI use** | 1.04 (0.87-1.23) | 0.71 | 1.07 (0.83-1.39) | 0.61 | 1.17 (0.97-1.40) | 0.12 | 1.05 (0.76-1.43) | 0.78 |
| **Myeloablative Conditioning** | 1.07 (0.94-1.22) | 0.35 | 1.04 (0.86-1.26) | 0.71 | 0.93 (0.80-1.08) | 0.35 | 0.84 (0.66-1.06) | 0.14 |
| **Donor-Patient CMV positivity (Ref:** Neg-Neg**)** |  |  |  |  |  |  |  |  |
| Neg-Pos | 0.96 (0.80-1.17) | 0.70 | 0.85 (0.64-1.12) | 0.26 | 1.08 (0.88-1.33) | 0.48 | 0.97 (0.68-1.38) | 0.85 |
| Pos-Neg | 0.89 (0.71-1.11) | 0.31 | 0.74 (0.53-1.03) | 0.081 | 0.81 (0.62-1.05) | 0.11 | 0.91 (0.60-1.38) | 0.65 |
| Pos-Pos | 0.93 (0.79-1.08) | 0.34 | 0.86 (0.69-1.07) | 0.18 | 0.91 (0.77-1.08) | 0.29 | 1.02 (0.77-1.35) | 0.91 |
| *1* HR = Hazard Ratio, CI = Confidence Interval | | | | | | | | |
| *2* *p<0.05; **p<0.01; ***p<0.001 | | | | | | | | |
